# Toward a Future Orientation: A Supportive Mental Health Facility Environment

**DOI:** 10.1177/19375867231221151

**Published:** 2024-01-23

**Authors:** Anne Hagerup, Helle Wijk, Göran Lindahl, Sepideh Olausson

**Affiliations:** 1 Institute of Health and Care Sciences, Sahlgrenska Academy, University of Gothenburg, Gothenburg, Sweden; 2 Inland School of Business and Social Sciences, Inland Norway University of Applied Sciences, Inland Norway; 3 Department of Quality strategies, Region Västra Götaland, Sahlgrenska University Hospital, Gothenburg, Sweden; 4 Department of Architecture and Civil Engineering, Center for Healthcare Architecture, Chalmers University of Technology, Gothenburg, Sweden; 5 Division of Construction Management/Center for Healthcare Architecture, Department of Architecture and Civil Engineering, Chalmers University of Technology, Sweden; 6 Department of Anesthesiology and Intensive Care/Sahlgresnka, Sahlgrenska University Hospital, Gothenburg, Sweden

**Keywords:** Access to nature, building design, mental health facility, qualitative research, supportive environment

## Abstract

**Background:**

The provision of supportive environments is essential in clinical and environmental psychology. Mental health disorders are a major issue, and the experience of being at a mental health facility is affected by numerous factors related to the building’s design.

**Aim:**

The aim of this study is to explore the expectations of a mental health facility planning group regarding the potential impact of a supportive design on patients’ mental health and staff’s therapeutic practices when planning and designing a new mental health facility.

**Methods:**

The new mental health facility is a case study and data were collected through qualitative in-depth interviews with nine participants and analyzed using a thematic analysis. The participants came from a mental health facility planning group in a new mental health facility in Norway.

**Results:**

The overall expectation of the new building was related to a future orientation to support patients’ mental health and therapeutic practices. Three main themes were identified: toward a future orientation, supportive building design, and work environment.

**Conclusions:**

Supportive environments are expected to influence patients’ mental health and staff’s therapeutic practices, including providing options for novel treatment needs in contrast to older and more outdated buildings that are perceived as hindering appropriate treatment conditions.

Most patients experience considerable stress when admitted to mental health facilities, and stressful experiences can have a negative influence on psychological and behavioral aspects. Because supportive designs promote well-being and are designed to foster coping with stress, such designs for mental health facilities are needed.

Mental health disorders are a major health problem worldwide and are related to severe distress, functional disabilities, and heavy economic burdens. According to the World Health Organization (2023), around 20% of the world’s children and adolescents have a mental health condition, with suicide being the second leading cause of death among 15–29-year-olds globally in 2019. Enhancing treatment conditions for people suffering from mental disorders will therefore benefit the individual suffering, their relatives, and society. A focus on planning and designing a physical environment that is expected to facilitate patients’ mental health, their conditions for treatment, and staff’s therapeutic practices should therefore be of importance.

In clinical and environmental psychology, the study of the physical environment is central to shed light on the quality of care for people with mental disorders, but historically, little research has involved the actual environment of these mental health facilities. Research from other fields was also imported into the discussion on the design and evaluation of mental health facilities, which prevented the development of purpose-designed methodology. Because of the wide variety of care options combined with inadequate funding for building design research on mental healthcare, the progression from experimentation to an evolved model of care was hindered (Chrysikou, 2013).

According to Ulrich (1991), an individual’s psychological well-being could be positively affected if that individual rated some elements of the physical environment as high in quality or attractiveness, which can enhance the individual’s self-esteem or self-image. In this article, a supportive environment refers to “environmental characteristics that support or facilitate coping and restoration with respect to the stress that accompanies illness and hospitalization” (Ulrich, 2001, p. 53) and takes its starting point from Ulrich’s (1997) theory of supportive design. Therefore, the concept of a supportive environment denotes a safe environment that is a setting in which both patients and staff can feel socially, emotionally, and physically safe and respected. Moreover, a supportive environment is considered to be a solid foundation for enhancing mental health and therapeutic practices, as there are also psychosocial and work environmental aspects to consider. Mental health relates to our thoughts, feelings, behaviors, and relationships with other people. Ulrich does not specifically address mental health facilities, but the focus on supportive environments in hospital in general is considered to have a significant impact also on patients with mental health disorders. Stefan Lundin has on the other hand specifically dealt with mental health facilities and is occupied, among other, with exploring how architecture can serve as a tool for faster and better rehabilitation (Caldenby et al., 2017). By focusing on how building design can improve treatment outcomes and patients’ well-being, he researches how the physical environment may play a role in supportive and therapeutic environments within mental health facilities (Lundin, 2009).

Most patients experience considerable stress when admitted to mental health facilities, and stressful experiences can have a negative influence on psychological and behavioral aspects. Because supportive designs promote well-being and are designed to support and foster coping with stress, this implies that supportive designs for mental health facilities are needed.

***Most patients experience considerable stress when admitted to mental health facilities, and stressful experiences can have a negative influence on psychological and behavioral aspects. Because supportive designs promote well-being and are designed to support and foster coping with stress, this implies that supportive designs for mental health facilities are needed.***

In sum, a supportive environment enhances positive relationships and communication. It is an environment that is experienced as safe, warm, welcoming, and having a relaxed atmosphere, in which relationships are built on trust and mutual respect. It concerns relationships between staff as well as between the staff/therapist and the patient. Communication is usually sensitive, patient-oriented, and respectful, even when it is busy or when staff are dealing with difficult situations (Commonwealth of Australia as represented by the Department of Health and Ageing, 2010). In this article, “the window of tolerance,” mindfulness, body-oriented techniques, and behavioral therapy are discussed in the section of theoretical standpoints as a way to understand from a clinical and environmental psychology perspective the way supportive environments may impact patients’ mental health and staffs’ therapeutical practices.

The present study is part of a larger project aimed at studying a new mental health facility as a case by exploring the meanings of a supportive environment and building design in mental health facilities from the perspective of a mental health facility planning group, psychologists, psychiatrists, patients, and staff. The aim of this article is to explore the expectations of a mental health facility planning group (hereafter called the “project group” for short) about the potential impact of a supportive design on patients’ mental health and staff’s therapeutic practices when planning and designing a new mental health facility. The mental health facility will be an in-patient clinic and have 70 patient rooms for adults and 10 patient rooms for adolescents aged from 12 to 18. There will be different departments containing acute and stabilized units in addition to open and closed units in general psychiatric care. This new mental health facility strongly encourages the therapeutic use of nature in the treatment of mental disorders, and this is a unique feature. Using the outdoors and nature in treatment, in accordance with bringing nature inside the building, is regarded as therapeutic by the project group. Thus, it has been important for the project group to facilitate access to nature.

## Previous Research

Research on issues related to the design of facilities and buildings is scarce in environments for mental health. Most research on healthcare facilities has focused on general care facilities, general issues, and work environmental issues (Weber et al., 2022). A review by Shin et al. (2021) suggests that a critical review of recent research is both timely and crucial for setting a future research agenda that includes a wider scope of clinical areas. New methods of conducting research and planning can help to further develop a knowledge base that planners and designers can use to develop spaces that support and even promote positive mental health. According to Shin et al. (2021), green places have been found to improve mental health, and findings similar to another study that showed that landscape architecture and gardens could reduce depression and increase mental health (Abbasian et al., 2020). Likewise, Rehn et al. (2022)—who conduct research on design strategies to influence health behavior—claim that mindfulness and relaxation are powerful techniques for increasing mental health by drawing people’s attention to design interventions that guide a person’s view toward, for example, natural scenery. Furthermore, 13 key themes were found in their review of the research literature on the effects of the architectural designs of mental health facilities on users. This review had the objective of presenting an updated review of the literature across health and architecture. The review focuses on mental healthcare and design in order to make a contribution to this field and assist other researchers, architects, and clinical practitioners to support and improve mental health outcomes. The authors argue that the use of gardens is highly relevant to mental healthcare (Connellan et al., 2013). A summary of existing evidence in relation to positive distraction and nature has shown a positive effect arising from direct access to nature and a view of nature, films or photographs of nature, nature sounds, or VR environments with nature elements. Research has addressed the effect of nature impressions on stress and anxiety, the experience of pain, anger, and aggression, increased well-being for patients, and reduced burnout for staff (Center for Healthcare architecture, 2021). Consequently, building designs related to mental health facilities should strive to maximize access to green spaces.

A systematic examination of peer-reviewed studies focusing on the built environment and mental disorders proposes that environmental design can trigger or reduce mental disorder symptoms. However, there is a lack of design research related to different types of mental disorder prevention and intervention. It can be concluded that studies on built environments should focus on improving or preventing the symptoms of all types of mental disorders through the design of physical environments (Aljunaidy & Adi, 2021). A systematic review on mental health facility design and fostering positive social interaction found evidence that should inform the design of new mental health facilities and the retrofitting of existing ones. These included, among others, providing a homelike environment, ensuring a good balance between private and shared spaces for patients and staff, and introducing plants into the environment (Jovanović et al., 2019).

The Safety and security, Competence-wise, Personalization and choice (SCP) model is a three-dimensional comprehensive theoretical model for understanding, profiling, and evaluating mental healthcare architecture and a grid for researching. By using the SCP model, these topics could be improved. The model puts together the dialectics behind mental health and addresses the relation of the facility to the individual in terms of the facility’s ability to cater to patients’ needs. These needs include those expressed by the jurisdictional, medical, and psychosocial models of care that mental health services historically incorporated and still play a dominant role in the mental healthcare regime and mental health building design (Chrysikou, 2013). According to Chrysikou (2013), it is not uncommon for mental health facilities to perform poorly in terms of patients’ social reintegration and developing institutional environments, despite being praised by the architectural media or even awarded.

Ward Atmosphere Scale (WAS) has been the most widely used instrument for measuring the psychosocial climate of inpatient units, thus focusing on different dimensions than the SCP model. According to the researchers, the climate of psychiatric wards influences patient satisfaction, improvement, and dropout rates (Røssberg & Friis, 2003a). A revised study replicated the findings that inpatients with psychosis prefer a high level of support, practical orientation, order, and organization. The study indicates that inpatients prefer a high level of involvement and, to some extent, a low level of staff control. It could be argued that a lot has changed during these years in mental health facilities. However, most of the psychiatric wards were rated in the 1990s, and when the researchers limited the analysis to mental health facilities evaluated after 1990, they essentially received identical results to the researchers previously studies (Røssberg & Friis, 2003b).

A study of service quality and clinical outcomes in mental health rehabilitation services was conducted in England. This study found that, by using standardized tools, all aspects of service quality were positively associated with service users’ autonomy, experiences of care, and therapeutic milieu, but there was no association with quality of life. Furthermore, quality of care is linked to better clinical outcomes in people with complex and long-term mental health problems. Thus, investing in the quality of care is likely to show real clinical gains (Killaspy et al., 2013). Another study focusing on patients’ experiences of place and space after relocation to evidence-based design forensic psychiatric hospitals revealed that purpose-built environments can support everyday living and well-being and create comfort. This was considered highly therapeutic by the patients (Olausson et al., 2021).

Setola (2014) has conducted multidisciplinary research in hospital facilities aiming to provide criteria for the evaluation and design of space and organizations in relation to regional and national policies on health in Italy. The research addresses the study of the relationship between the protection of the right to health and the design and use of hospital spaces. The basic idea behind the model is to spatialize flow to identify the procedural pathway involving the patient in the physical space of the hospital. The research results include knowledge articulated in a model, process data sheets, and indicators for the evaluation of the flows in hospitals. Even though the data are collected in hospitals, the focus is on mental health and design; thus, the findings may be relevant for mental health facilities.

We argue that the aforementioned research gives reason to anticipate that the findings are also true for similar physical environments within mental health facilities. Furthermore, autoethnographical data show that building interiors can be considered a metaphor for an inner dimension of the self. It is not uncommon for spaces in hospitals to be experienced as representing an institutional and technical dimension (Grignoli, 2021), and every aspect that supports the patients’ sense of self should be welcomed and strived for.

Moreover, what could be called psychologically “hard” building design—in this article, referring to an environment focusing on function and security—may be functionally effective but not perceived as supportive; it may also be potentially stressful for patients, visitors, and staff (Ulrich, 1991, 1999). Research has linked design that has not considered the needs of patients to have a negative impact on patients’ well-being, patients lacking social support from significant others, and, in some cases, having a negative impact on the healing processes (Ulrich, 1991). A supportive design approach emphasizes and includes opportunities in the environment that can calm patients, reduce stress, and strengthen coping resources (Ulrich, 1991, 1999). This is achieved by addressing environmental characteristics that research has found to be stressful or that may have direct negative effects on treatment outcomes. Three components were proposed for creating a supportive healthcare environment: fostering control (including privacy), promoting social support, and providing access to nature and other positive distractions (Ulrich, 1991). Andrade and Devlin (2015) tested Ulrich’s theory of a supportive environment, and the results of their study confirmed this theory. The participants experienced significantly less stress in situations, where all three components were present or only positive distraction and social support.

A supportive design approach emphasizes and includes opportunities in the environment that can calm patients, reduce stress, and strengthen coping resources. This is achieved by addressing environmental characteristics that research has found to be stressful or that may have direct negative effects on treatment outcomes.

***A supportive design approach emphasizes and includes opportunities in the environment that can calm patients, reduce stress, and strengthen coping resources. This is achieved by addressing environmental characteristics that research has found to be stressful or that may have direct negative effects on treatment outcomes.***

## Theoretical Standpoints

The theoretical standpoints of this study come from environmental psychology and clinical psychology. Environmental psychology has dealt with three major themes that have been overlooked by different areas in psychology: (1) the need to understand behavior in context: people in specific places, (2) the reciprocal relationships between people and places, and (3) the need to be interdisciplinary (Clayton & Saunders, 2012).

The study therefore seeks to understand how the project group expects the built environment to affect patients’ mental health and staff’s therapeutic practices. Following this reflection, the environment is essential for how individuals feel comfortable and safe. To describe the best state of arousal or stimulation in which individuals are able to function and thrive in everyday life, “the window of tolerance” can be used. The window of tolerance is a concept originally developed by Siegel (2015) to describe the optimal zone of the autonomic “arousal” for an individual to function in everyday life. Positive distraction or external stimuli is widely used to regulate activation. When individuals operate within this zone or window, they can effectively manage and cope with their emotions.

When admitted to a mental health facility, patients may experience stress, crowding, boundary-setting issues, or remembering a traumatic memory or triggers, and this could cause patients to be pushed out of their window of tolerance. Even seemingly minor stressors can cause an individual to dissociate, get angry, or feel anxious, thereby leading to dysregulated states of hyper- or hypoarousal; thus, they are outside the window of tolerance. Because of this, it is important to offer patients environments that are as supportive as possible, so that patients will more easily manage to be in their window of tolerance. They will then become more available for therapy. External focuses or positive distractions draw attention to something outside oneself, and this may be effective in therapy, both with regard to building a therapeutic alliance and the possibility of regulating the patient when he or she is emotionally overwhelmed. Techniques from mindfulness (Kabat-Zinn, 2013), body-oriented techniques (Ogden & Fisher, 2015), and cognitive behavioral therapy (Linehan, 2014) include the use of external focus or positive distractions. Views of nature and nature elements may lead to faster recovery as a form of richer external stimuli or positive distraction (Center for Healthcare architecture, 2021). Given the theoretical standpoints, it is reasonable to question how this knowledge is incorporated and taken into consideration when planning and designing a new mental health facility that achieves supportive environments to facilitate patients’ mental health and staff’s therapeutic practices.

## Methods

### Design

The study used a qualitative approach with interviews with key participants in a project group who were engaged in planning and designing a new mental health facility. The study is based on the thematic analysis methodology described by Braun and Clarke (2006). This method was chosen to gain a deeper understanding of the participants’ expectations of the potential impact of a supportive design on patients’ recovery and staff well-being when planning and designing a new mental health facility. The members of the project group had in-depth knowledge of the new mental health facility and could provide thick descriptions. In-depth interviews were used to explore the experiences of the participants and the meanings they attributed to these experiences (Tong et al., 2007). Open-ended questions in one-on-one interviews were also used to encourage participants to talk about issues pertinent to the research questions. The research questions that guided this study are as follows:

1. How has the project group understood and applied the concepts of supportive environment to the project studied?
2. What opportunities are reflected upon when planning and designing this new mental health facility?

## Setting

### Intentions for the Case called Nybygg Psykisk Helse Kristiansand

Case studies focus on specific units of analysis, called a case, and are characterized by this rather than the methods used for data collection or analysis (Willig, 2013; Yin, 2018). Cases focus on a specific real-world situation and aim to provide a detailed examination of the subject matter and to analyze, interpret, and draw lessons from the particular case and thereby helping researchers and practitioners to gain insights into complex issues (Yin, 2018). In this study, Nybygg Psykisk Helse Kristiansand (New Mental Health Facility Kristiansand, Norway), hereafter called NPK for short, is a case and case studies are typically considered within its context. This study was conducted at a new Norwegian mental health facility under construction. One of the core ideas behind the building was the use of nature in and around it, and a strong focus on the therapeutic use of nature is a key feature within the facility. According to the project description, the building aims to be a facility in Norway that promotes future-oriented treatment options within mental health for adults and young people. In the preliminary project report, future-oriented treatment refers to having flexibility about future operations and needs not yet known, allowing changes in the form of treatment and utilization of areas for different patient groups, and having opportunities to handle new tasks that may arise. Furthermore, the project description states that the building aims to enable continuous development of what is offered to patients, their interactions with other parts of the health service, and ensure that patients receive the professionally best and most effective course of treatment on offer. The facility possesses an attractive appearance that promotes mental health, and it aims to provide employees with a good physical working environment that contributes to efficient work processes.

The main landscape architectural approach must ensure a connection between the landscape, building, and surroundings. In the concept work, the Norwegian Government (2017) calls for the use of nature and the environment as an integral part of the treatment. The starting point is to have the best possible knowledge of population development, disease development, and medical professional development during planning and construction. The new building will be completed for patients in spring 2023 and will contain a total of 80 patient rooms with 24-hours treatment available. There will be one section for adolescents aged 12–18 and one section for adult patients aged 18 and older. There will be both acute and intermediate treatment areas for general psychiatric care.

### Building Design of the Case Called Nybygg Psykisk Helse Kristiansand

**Figure 1.**
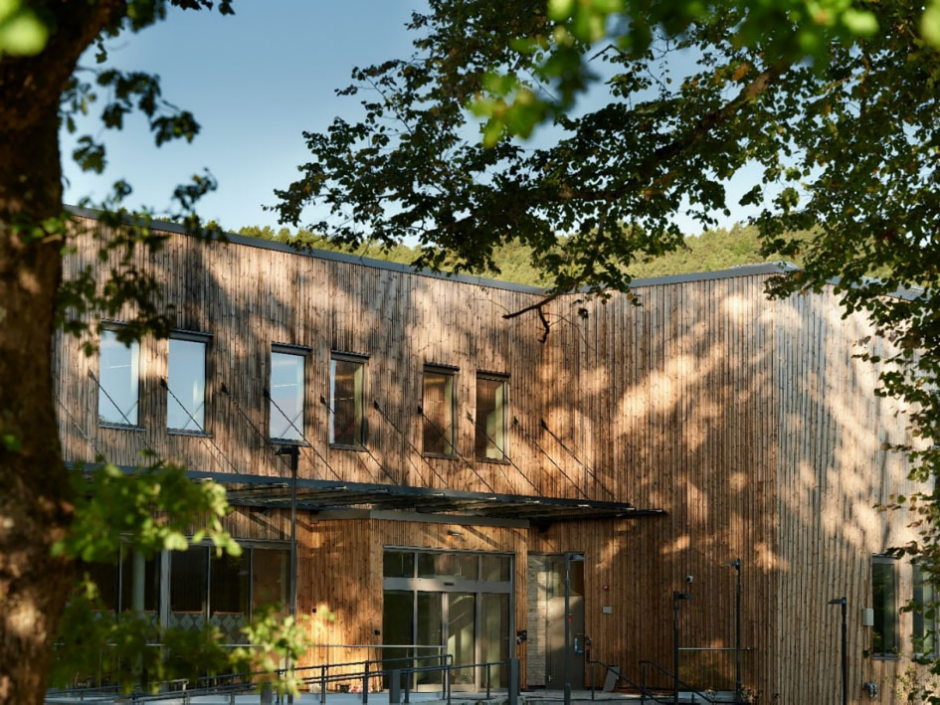
Main entrance. Photograph: Marcel Tiedje.

**Figure 2.**
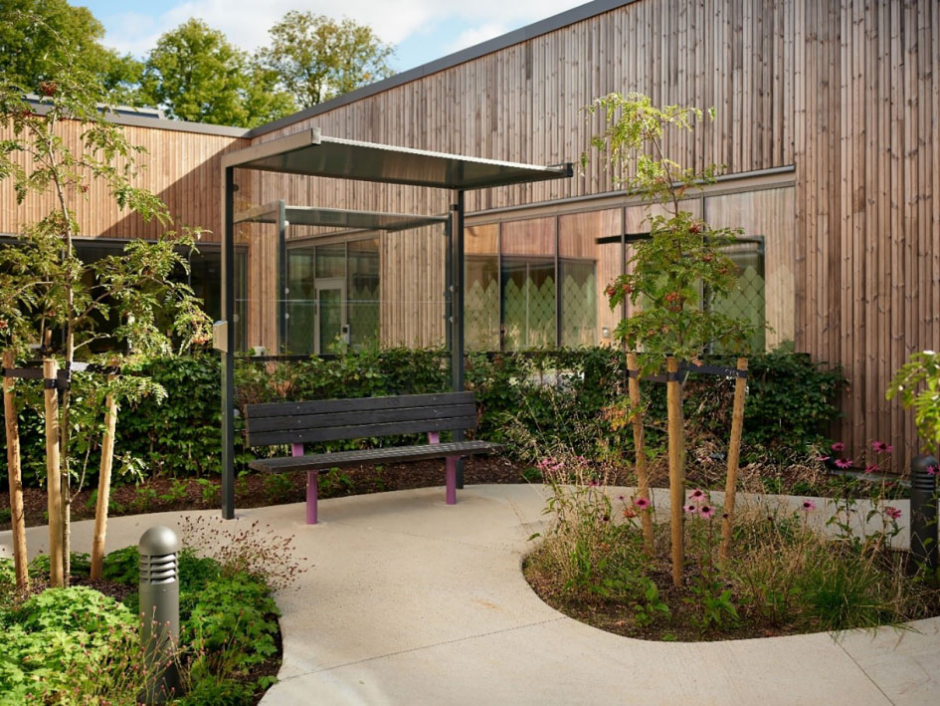
Inner courtyard. Photograph: Marcel Tiedje.

The new mental health facility will be located in a rural area surrounded by nature in the general hospital area. Contact between nature and the building is considered important; thus, circadian rhythms, weather conditions, and elements of nature will be integrated into the building. The walls in the inner courtyards will be transparent glass that provides daylight into the units and creates a strong connection between the corridors and the outside areas. The lushness of the forest will be drawn into the courtyards and between the wings. The building’s access to outdoor areas, courtyards, and screen gardens will be easy; a number of smaller sheltered gardens will be directly connected to the building’s indoor functions. These gardens will be an essential part of a health-promoting outdoor area, as they will offer patients the opportunity to experience nature and have fresh air. The courtyards must also have health-promoting functionality, have good aesthetic qualities, and prevent patients from acting out. The surroundings must therefore be easy to “interpret,” so that patients can feel safe. The gardens will be shielded from the public, with an enclosed wall and plants. Please see Figures 1 and 2.

## Participants

The participants in the project group consisted of clinical psychologists, psychiatrists, leaders, engineers, architects, and landscape architects. The sample consisted of key participants (Tong et al., 2007) because the participants in the project group who were involved in the new mental health facility were considered to share particular characteristics. Therefore, they have the potential to provide rich, relevant, and diverse data. They were chosen for their firsthand knowledge of the new project and thus for their ability to provide in-depth knowledge about the topic of interest. Some of the participants (those who were not architects, landscape architects or entrepreneurs) did not have experience with design planning; however, they had extensive experience with clinical work and/or leadership/general project planning.

## Preparation

The user–coordinator within the project group suggested members from the project group to be participants in the study to ensure a wide selection of participants—for example, participants from different professions and thus with different expectations of the mental health facility under construction. The user–coordinator wrote an e-mail to the group members and asked them to respond to the first author (interviewer) if and when they were asked to participate in the study. Based on these suggestions, a total of 14 group members were invited by the first author via e-mail to provide information about the objective of participation in the study. A total of nine participant agreed to take part in the study and five persons declined. Two participants with the user perspective (i.e., patients, previous patients, and/or relatives) who were part of the project group were invited to take part in the study. However, one person could not attend because of sick leave, and the other person could not attend in the period during which the interviews were conducted. The first author also participated in a project meeting before the interviews took place to inform the participants about the study and answer questions that they might have and to provide information about informed consent, the reasons for conducting the research, and that it was possible to withdraw at any time without supplying a reason for withdrawal. Nine participants agreed to participate, and five declined, did not reply, or could not participate during the period in which the interviews were collected. None of the participants withdrew from the study after agreeing to participate and providing informed consent.

An interview guide was developed based on the research questions and aims of the study. The objective was to explore the expectations of a project group regarding the potential impact of a supportive design on patients’ mental health and staff’s therapeutic practices when planning and designing a new mental health facility.

## Data Collection

The participants were encouraged, through open-ended questions, to talk about the expectations pertinent to the research questions. The interviews began with an open-ended question regarding the participant’s role in the project. Subsequent questions were based on their expectations of the impact of the healthcare environment on the mental health facility, and the focus was particularly on what the participants considered supportive. This focus was not communicated to the participants before the interviews took place. Please see attached interview guide.

Handwritten notes about relevant findings were made, and follow-up questions were asked during the interviews. All interviews were performed individually, except for one interview with two participants (numbers 7 and 8) who were colleagues, as they wanted to participate together or not at all. Eight interviews were conducted in person/physically, and one interview was conducted via Zoom. The length of the interviews ranged from 27 to 64 min. The average interview time was 44 min, and they were audio-recorded and transcribed verbatim by the interviewer. Most of the interviews took place at The Outdoor Care Retreat (a wooden cabin located at the hospital premises), as it was considered to supply a natural frame being located in nature with natural materials, rich exposure to daylight with big windows, and a view of nature. However, not all participants wanted to travel to The Outdoor Care Retreat for practical reasons and due to a shortage of time. Five interviews took place in The Outdoor Care Retreat, three in the participants’ office, and, as previously mentioned, one interview was digital on Zoom. Only the interviewer and the participant were present.

## Data Analysis

The interviews were analyzed using thematic analysis (Braun & Clarke, 2006). Thematic analysis is a method for analyzing and reporting patterns and meanings within interview data. According to Braun and Clarke (2006), thematic analysis is administered by a six-step process: (1) familiarization with the data by transcribing, reading, and rereading initial ideas, including notes on the general data set; (2) systematic identification of codes when features of the data across the entire data set could be relevant to identify potential subthemes/themes and when they are considered to be related to the aim of the study. Codes identify a feature of the data that appears interesting to the analyst and refer to the most basic segment or element of the raw data or information that can be assessed in a meaningful way regarding the phenomenon. The codes consisted of short phrases to attempt to capture the essence of the text relating to the research questions and lay the foundation for the thematic development of the analysis; (3) collation and relating codes to potential subthemes and themes and gathering data that are relevant to each potential theme, including clustering similar codes together. The data were critically reviewed and revised until a final pattern emerged; (4) check whether the themes functioned in relation to the coded items and the entire data set and whether the themes had a distinct essence or central organizing concept that addressed the research questions; (5) continued analysis to refine the specifics of each theme and the overall story of the analysis by naming each theme. This is to strive to ensure conceptual clarity and mapping in the final report, including the researcher’s commentary on the data; and (6) final analysis and focus on compelling extracts as examples relating to the study’s research questions. This last stage also included choosing citations from the participants and themes connected to theory and empiricism. To secure rigor, validity, credibility, and transformability, the strategies functioned to explain the context and the case as thoroughly as possible, so that the reader might judge the validity and credibility of the analysis and results and the transformability of the results to similar situations (Kvale & Brinkmann, 2019). The analysis was performed to ensure that the codes and themes mirrored the meaning of the text. Reflective questions, such as, “How do I know that this is the meaning of the text? Could it be something else?” were included. The analysis followed the consolidated criteria for reporting qualitative research, a 32-item checklist for interviews (Tong et al., 2007).

## Preunderstandings

The analysis was conducted by the first author with feedback from the other researchers. The authors have preunderstandings that may have influenced this research (Kvale & Brinkmann, 2019; Silverman, 2017). However, the authors systematically challenged their respective preunderstandings through discussions and reflections during the research process. Author AH is a female clinical psychologist and environmental psychologist, currently doing a PhD in healthcare sciences, who has been working with patients for several years and has extensive experience conducting interviews. Author HW is a professor and senior consultant registered nurse and a researcher with extensive experience in environmental healthcare studies. Author GL is a professor and an architect who researches the planning and design of healthcare environments, and author SO is an associate professor and a specialist nurse with extensive experience in qualitative methods and research within the impact of environment on healthcare settings in other mental healthcare settings.

## Ethical Considerations

All participants were informed, in written format and orally, about their right to discontinue their participation without further explanation. They were also given contact information for the researchers responsible and their institutions. All data were handled with confidentiality. During the execution of this study, no risks were identified for the person involved. All participants gave written consent to participate.

The study was performed in accordance with the Norwegian Center for Research Data to ensure the subjects’ anonymity and data protection (privacy). The project was submitted to the Regional Committees for Medical and Health Research Ethics (REK), but REK concluded that approval from REK was not required to carry out the project, as the project was outside the scope of health legislation.

## Findings

The nine participants interviewed were from 38 to 65 years old, with an average age of 50.8 years, with five females and four males. Further demographic characteristics of the participants are presented in Table 1.

**Table 1.** Participants Characteristics.

**Participant number**	**Gender**	**Age Span**	**Professional Background**	**Duration of Interview**	**Location**
**Participant 1**	Male	55–65	Psychiatrist	46.11	Office
**Participant 2**	Female	55–65	Psychologist with clinical Speciality	39.53	The outdoor care retreat
**Participant 3**	Male	35–45	Entrepreneur	27.26	Office
**Participant 4**	Female	45–55	Psychologist with clinical speciality	33.55	The outdoor care retreat
**Participant 5**	Female	45–55	Clinical sociologist	50.46	Office
**Participant 6**	Male	35–45	Coordinator	39.04	The outdoor care retreat
**Participant 7**	Female	35–45	Architect	49.47	The outdoor care retreat
**Participant 8**	Female	35–45	Landscape architect	49.47	The outdoor care retreat
**Participant 9**	Male	55–65	Architect	63.56	Zoom
**Average**		50.8		44,27	

Table 2 presents the exploration of the nine participants’ expectations of the impact of the healthcare environment on patients’ mental health and staff’s therapeutic practices when planning and designing a new mental health facility, with a focus on what they considered the potential impact of a supportive design. The findings are described in three main themes and 10 subthemes.

**Table 2.** An Overview of the Findings.

Main Themes	Subthemes
Toward a future orientation	Novel design solutions New dimensions to therapeutic practices Professional expectations
Supportive building design	Autonomy, integrity, and normalization Flexibility for individual needs Nature-inspired design Prioritizing individuals and their dignity
Work environment	Promoting well-being for staff Greater safety and security Enabling a therapeutic alliance

## Toward a Future Orientation

The participants’ overall expectations of the new mental health facility with novel design solutions could support new treatment strategies and meet the future needs that older and more outdated buildings in the hospital area could not offer. According to the participants, treatment options include opportunities for patients’ seclusion outdoors in gardens instead of inside a room. Since patients are mainly allowed to go outside in safe atrium gardens as often as they like, boundary-setting situations may diminish or be reduced. Subsequently, this could lead to less acting out and increased safety and security for the patients themselves, staff, and other patients.

In addition, the participants reflected that this novel design could add new dimensions to therapeutic practices. For example, daylight, birds, sounds, smells, seeing the seasons, looking out the window, and being outdoors in nature were believed to provide metaphors to use therapeutically. This may produce other types of conversations in therapy, as nature becomes an agent for metaphors using other denominations and themes that neither the patient nor the therapist has previously considered.

The new building was also described as providing professional expectations and opportunities different from older and outdated (aversive) buildings. Treatment options may also be more varied, as the building provides flexibility for the patients and therapists, as the therapeutic styles and methods are different. Flexibility and varied treatment options were believed to increase the potential for better treatment outcomes. It referred to older and traditional buildings that could not provide support and opportunities in the same way due to building design limitations. Additionally, the professional expectations consisted of the hospital environment in a new way with new solutions for the interior and exterior and strongly pursuing the idea of supportive design strongly.

The new building was also described as providing professional expectations and opportunities different from older and outdated (aversive) buildings.

***The new building was also described as providing professional expectations and opportunities different from older and outdated (aversive) buildings.***

## Supportive Building Design

One of the ideas behind the new mental health facility was enabling opportunities for patients to choose for themselves whether they stayed in common areas, went outdoors, or retreated to quiet zones and “caves” where they could find peace and silence. These aspects were seen as enhancing the protection of an individual’s autonomy and integrity and contributing to a normalized state of being. For example, patients do not have to ask for an escort if they want to go out because they are in a safe but positive environment in which they can choose for themselves how they want to use it.

One of the ideas behind the new mental health facility was enabling opportunities for patients to choose for themselves whether they stayed in common areas, went outdoors, or retreated to quiet zones and “caves” where they could find peace and silence.

***One of the ideas behind the new mental health facility was enabling opportunities for patients to choose for themselves whether they stayed in common areas, went outdoors, or retreated to quiet zones and “caves” where they could find peace and silence.***

Moreover, the participants highlighted the importance of inviting people into the new building, the work that is being done there, the aim of reducing stigma about mental disorders, and openly showing what they are doing. Often in mental healthcare, the public comes to a closed and locked door. This is in contrast to what is experienced in a somatic hospital, where doors are usually open, and the public are allowed in. In this new building, there is going to be a generally publicly accessible vestibule and cafe, as in somatic hospitals. The emphasis is on the fact that ordinary people who mentally strive are staying there for a period.

The participants’ expectations included reflections on the fact that, when people are in hospital, their choices are usually limited. This makes it important to be able to offer patients various options, as patients with mental disorders are a group with many different needs. The new mental health facility is designed to be flexible and to meet the individual’s needs. Additionally, there will be specific areas for the patients to choose to participate in activities like nature experiences, gymnastics, music, art therapy, and ball games. Flexibility also provides varying opportunities, since people cannot be standardized.

The project group has consciously worked with the elements they could regulate and vary, and the interior architect has used colors from nature inside the building—that is, the idea of nature-inspired design. Natural materials, wood, daylight, air, and natural views eliminate institutional features, so the interior can be robust but still beautiful. Nature is close by, and because the outside–inside relationship is important, the patient premises are on the ground level. The participants emphasized the importance of both the building’s new design and that nature was integrated in and surrounded the building. Several participants felt that the old buildings were not supportive but anti-therapeutic. They continued that the old buildings resembled something other than what was desired from a good treatment building. The new building must be one that welcomes people with an entrance worthy of those who enter it. It must be nice, open, inclusive, and respectful, and the landscape architect has designed it as a garden, such as what people have at their homes, so patients can feel comfortable and safer and thus have reduced stress. This means that the building should reflect the values/ideologies of what is known to be supportive, thus nurturing and fostering caring practices.

Moreover, the new hospital will prioritize individuals and their dignity by offering a welcoming atmosphere, even to individuals who have not necessarily chosen to be admitted to the mental health facility. The first impression at the entrance was of coming into a garden with grass and flowers. The participants also highlighted the symbolism of a new and well-designed building as a priority for the professional field, thus prioritizing patients and mental health. In the same way that poor investment in the building creates a feeling of a lack of prioritization, a well-presented building that makes people smile when they enter is a way of prioritizing patients.

## Work Environment

The potential positive benefits of nature are believed to make it easier for staff members to recover from mental exhaustion because nature will be easily accessible to the staff. The participants reflected on this fact and considered it to be promotive to the staff’s well-being. Staff may be working in the environment every day for several years, so the well-being of staff also influences the quality of care for patients.

The building design will make everyday life easier for employees with greater safety and security because of straight corridors that promote lines of sight and two digital projects: a medicine cabinet to increase safer medicine management and presence sensor/monitoring technology (StaySafe) to monitor patients when they are alone in their rooms. There will be no long transport stages—that is, long corridors—but rather fast track between departments to ensure time with patients, and this will make cooperation easier. The opportunity to build professional relationships and gain and give support to colleagues will be improved compared to the old premises. This is because all departments and offices are built under one roof, and several departments are located together. Pooling expertise is made possible and easily accessible. Therefore, the new building is organizationally different from the existing premises.

The potential positive benefits of nature are believed to make it easier for staff members to recover from mental exhaustion because nature will be easily accessible to the staff.

***The potential positive benefits of nature are believed to make it easier for staff members to recover from mental exhaustion because nature will be easily accessible to the staff.***

Some of the participants expected that the opportunity for cooperation would be safer and easier because of the colocation of different professional disciplines and patients (adolescents, adults, and the elderly with different diagnoses) in the same building. This may entail holistic thinking, which is beneficial for both patients and staff.

Will the building and its design enable a therapeutic alliance between the therapist and the patient so that the patient feels calmer and more secure? To achieve this alliance, the rooms used for therapeutic conversations must invite confidentiality, so that other people are unable to see or hear what is going on in the room. If patients feel insecure or unsafe, they might withhold important information during therapy sessions. Please see Table 3 for participants’ quotes related to each theme and subtheme.

**Table 3.** Participants’ Quotes Related to Each Theme and Subtheme.

Main Theme	Subtheme	Quote
Toward a future orientation	Novel design solutions	The main idea is to have a building with premises and outdoor areas that support the latest treatment methods…and treatment opportunities within mental health care to a much greater extent than what we have in the current situation. (Participant 6, clinical staff)
Supportive building design	Autonomy, integrity, and normalization	Our way of thinking rests on the very basic concept of recovery. We have tried to stick to that. It is not a place to live for the rest of your life, but a place to recover. (Participant 9, facility planner)
Supportive building design	Nature-inspired design	And that we use such large open glasses in buildings, and that we have these outdoor spaces that are built up with a lot of nature, I think that both the daily rhythm of nature will also come into the building to a large extent. Both with regards to the rhythm of the day, but also the seasons, how these outdoor spaces change. From winter and spring and summer and fall. (Participant 6, clinical staff)
Toward a future orientation	New dimensions to therapeutic practices	And also that you can use it more psychotherapeutically in the sense of everything from how you can get associations that are completely different outside on a nice path than inside a room, and work further with sensory impressions…//. There is something there that is too little explored and used in traditional treatment. (Participant 1, clinical staff)
Supportive building design	Autonomy, integrity, and normalization	I think that architecture that explodes some of those boundaries and invites that they [patients] may come from a farm in the countryside or the archipelago, that is, things like that that are their own universe. That we don’t destroy or think that we own their imaginary world by forcing them into an ordinary 24-hour post…Some come with a dissolved self and that we then can offer some barriers to structure, and if it [the building] is too open, then will it be a threat. (Participant 1, clinical staff)
Work environment	Enabling a therapeutic alliance	You have to think about hierarchy, a dosage, in relation to when you are very ill, there are some things that should be limited, but as you get well, you can turn up the stimulus, opportunities for social contact. And that is fundamental. It is not a house; it is a series of possibilities. (Participant 9, facility planner)
Work environment	Greater safety and security	So I hope that everyday life will be easier. Greater degree of security as an employee in the building here. We have worked with lines of sight, the fact that you don’t have alleys and places you can hide away. But that you, as an employee, have sight lines and straight corridors, that we succeed in making everyday life safer. There has been a lot of focus in the patient area then. No long transport stages. (Participant 3, facility planner)
Work environment	Promoting well-being for staff	It was very important that everything should be on one level, so you didn’t have to jump up and down all the stairs and that you had a uniform…Yes, that you are united and that is perhaps the most important thing for us who has worked on the other units [old building premises] for all years. That we are together, because we can see that there is a completely different therapeutic culture that has brought everything together in one building. It is easy for the therapists to discuss, go in and discuss, seek help from each other. A completely different culture, which is also open. (Participant 2, clinical staff)
Supportive building design	Prioritizing individuals and their dignity	There is something about the signal effect to all users, there is important work being done here, isn’t it? The importance of that should not be underestimated. (Participant 5, clinical staff)
Toward a future orientation	Professional expectations	I have expectations that the building will break down those barriers, and that it contributes to us…the general public, gaining a greater understanding of what it is like to be admitted to a psychiatric hospital ward. (Participant 6, clinical staff)

## Discussion

This case study enabled the exploration of the project group’s expectations of the potential impact of a supportive design on patients’ mental health and staff’s therapeutic practices when planning and designing a new mental health facility. Prominent findings were that almost all participants believed that there would be many therapeutic opportunities available in the new mental health facility compared to the old premises and that participants were concerned about future orientations. Moreover, the study explored how supportive environments were understood by the project group and shed light on the opportunities that were reflected on when planning and designing the new mental health facility.

In this study, we found that the project group expressed how the new mental hospital was oriented toward the future by offering novel design solutions and new dimensions to therapeutic practices and professional expectations. By focusing on a supportive environment and nature so strongly, as this is a key feature within the new facility, the project group indicated that the facility was future oriented compared to old buildings. Even though it is not clearly stated what “future orientation” consists of, their expectation is that the new facility will offer “something more” than the old buildings. Their expectation of the physical environment’s impact on mental health is in line with a systematic study of peer-reviewed literature on architecture and mental disorders. The study proposes that a physical environment design can trigger or reduce mental disorder symptoms. However, there is a lack of knowledge on how this has been integrated into different types of mental disorder prevention and intervention (Aljunaidy & Adi, 2021), and thus, it seems important to shed light on this topic. Another review, published two decades ago, proposed a guideline for creating a supportive healthcare environment based on three components: fostering control (including privacy), promoting social support, and providing access to nature and other positive distractions (Ulrich, 1999). Additionally, the SCP model (Chrysikou, 2013), as discussed previously, highlights some of the same components as safety and security, competence-wise, and personalization and choice. The WAS (Røssberg & Friis, 2003a) also measures the psychosocial climate of inpatient units. Even if the dimensions are somewhat different from Ulrich’s model and the SCP model, a revised version of WAS (Røssberg & Friis, 2003b) indicates that inpatients prefer a high level of support, practical orientation, and order and organization, which is in line with findings from the previous two models discussed. Being able to support patients by giving them autonomy, integrity, and normalization—in accordance with flexibility for individual needs and prioritizing the individuals and their dignity—has been a major focus for the project group in this study and relates to how the project group understood the supportive environment. However, it remains to further investigate the outcome of all these features from the patients’ and staffs’ perspectives.

The use of nature both outdoors and indoors (i.e., nature-inspired design) was also considered by the project group as an important therapeutic element. The benefits of nature are well known. It can counteract stress, tiredness, mental fatigue, and compassion fatigue, according to both attention restoration theory (Kaplan et al., 1998) and stress reduction theory (Ulrich, 1983). In this study, there was a major focus on the supportive effects of nature, and this focus is in accordance with Shin et al.’s (2021) study that highlights how green spaces have been shown to improve mental health and suggests that healthcare site design should maximize access to green spaces. Furthermore, researches by Abbasian et al. (2020), Rehn et al. (2022), Connellan et al. (2013), and the Center for Healthcare architecture (2021) report therapeutic effects of nature on mental health, as discussed previously. The findings of this study indicate an awareness of the importance of the physical environment for treatment within mental healthcare and the creation of a space for therapy. The findings from the studies discussed are also in line with this study and the expectations of the project group regarding their work environment to promote staff well-being.

People become insecure when they have too little information and confused when they have too much information. This is according to the triangle of supportive environments (Bengtsson & Grahn, 2014). The rooms used for therapeutic conversations must invite confidentiality, so that the patient can feel secure and safe to openly discuss treatment and therapy with their therapist. According to Stern (1985), activating the parasympathetic nervous system and releasing the neurotransmitter oxytocin have been shown to improve the bond between the patient and the therapist. The project group highlighted that the new mental health facility could enable therapeutic alliances. Releasing oxytocin could make the patient feel calmer and more secure, which means that the patient can benefit from therapy in a more effective way than if they are stressed and anxious and thus potentially enhance their mental health. Furthermore, the therapist and staff may experience a sense of well-being when being with calm and secure patients, rather than when insecure patients are in danger of acting out verbally and/or physically. Thus, the participants in the project group expected greater safety and security.

The study presented contributes to knowledge on the process of integrating research findings into mental healthcare facilities planning when it comes to expectations of being future oriented and focusing on the therapeutic use of nature and nature-inspired design to support patients’ mental health. Furthermore, it has focused on the work environment and how these findings indicate that the staff’s therapeutic practices may be influenced.

## Methodological Strengths and Limitations

Although the study provided an understanding of what was considered supportive when planning and designing a new mental health facility, the physical environment is not the only factor that affects the impression of support and therapeutic outcomes. A limitation could be that all participants were very positive toward the new building and its design, and it is likely the participants could have been prejudiced. They also knew that the researcher was positive about and interested in this topic, which may have influenced their answers. According to Svartdal (2015), a challenge with data analysis could be that early information and first impressions are given greater value than is appropriate and may influence later information. The researchers were conscious of this and tried to minimize this risk by constantly reminding themselves of this phenomenon. The information could be difficult to handle in an objective manner, and interactions between the researcher and the participants could influence the results. Different researchers could arrive at different results, as the participants’ expectations are subject to interpretation by the researcher (Willig, 2013). Another challenge was that the interview guide was not tested through a pilot, as it was impractical at the time of data collection. Pilot testing could have altered the questions by shedding light on what questions were most important for obtaining answers to the research questions. Two participants with a user perspective (patients or relatives) were also asked to participate, but one person did not reply due to long-term sick leave, and the other person could not participate during the period when the interviews took place. Since these were the only two people with a user perspective, the study did not have a user perspective, which could have enriched the data sample and provided thicker descriptions. Interviews with patients at the new mental health facility will be conducted as part of a larger study when the building has been taken into use in spring 2023. Even though the project group was diverse in age, gender, profession, and roles, their views and perceptions could be homogeneous or polarized. The quality of the interviews might also have been affected by the fact that the project group participated in the same meetings and discussions. Thus, the group members might not be as open as they otherwise might have been if the data were sampled from different project groups. A final limitation was the small number of participants, and more participants could have provided more nuanced reflections. This was a qualitative study of what the project members understood the building would be like in the future, and there was no quantitative data to support the variables identified.

Some strengths and challenges of this study have been identified. One strength was that the project group seemed to have some understanding of how supportive environments could affect patients and staff. Additionally, the participants had nuanced reflections on different theoretical concepts related to supportive environments, even though they were not asked specifically about the concept of a “supportive environment” during the interview. Another strength that could compensate for the small sample size was the heterogeneity of the project group, which had a large variety of professional backgrounds. Both men and women from different age ranges were represented. All of the participants had in-depth knowledge about the project and could therefore give adequate reflections and thick descriptions.

Another element to consider was whether the environment during the interviews influenced the answers. Some of the participants were interviewed in a serene cabin located in the forest with a natural interior, daylight, and beautiful nature view, namely, The Outdoor Care Retreat, while others were interviewed in an office in older building premises.

The personality of the participants could also have had an impact on the reflections, as the responses of participants who did not want or did not have the time to go to The Outdoor Care Retreat could differ from those who prioritized going outdoors in nature as an important frame for conducting the interview. Finally, we need to stress that although the study provided an understanding of supportive hospital environments, the number of participants was small, and this was a qualitative study on how the project members perceived the building to be in the future and their expectations of this building. This study is a case study and because of a specific situation in a specific context at a specific time, the transferability must be questioned although comparison with experiences from other facilities can indicate patterns.

## Conclusion

The study found that, when designing a supportive mental health facility, opportunities for being future-oriented and flexible are important. It is necessary to keep the possibility of new treatment options in mind, as current research and clinical practices do not know how future treatment options will be. To have the possibility of individualizing patient rooms and tailoring treatment for each patient is essential.

Altogether, the study results provide knowledge on the expectations of a project group planning and designing a new mental health facility about the potential impact of a supportive design on patients’ mental health and staff’s therapeutic practices. This knowledge should be considered when designing new mental health facilities or refurbishing existing ones. As this study was conducted only in Norway, it is important to study the needs and experiences of other mental health facilities in different countries and with different patient groups to strengthen the basis for supportive design and its impact on treatment.

## Implications for Practice

- Being future-oriented includes having options for future treatment needs in contrast to older and more outdated buildings, and this provides the possibility to adapt the environment and physical features according to individual needs.
- Physical environments that are experienced as supportive may influence patients’ mental health and staff’s therapeutical practices in a health promoting way.
- The project group expressed that by offering novel design solutions, the new mental health facility will contribute to new dimensions in future therapeutic practices.
- The use of nature both outdoors and indoors (i.e., nature-inspired design) was also considered by the project group as an important therapeutic element.

## Supplemental Material

Supplemental Material, sj-docx-1-her-10.1177_19375867231221151 - Toward a Future Orientation: A Supportive Mental Health Facility Environment Supplemental Material, sj-docx-1-her-10.1177_19375867231221151 for Toward a Future Orientation: A Supportive Mental Health Facility Environment by Anne Hagerup, Helle Wijk, Göran Lindahl and Sepideh Olausson in HERD: Health Environments Research & Design Journal
