# Supplementary material for: Toward a Future Orientation: A Supportive Mental Health Facility Environment
Source: HERD. 2024 Jan 23;17(2):38–56. doi: 10.1177/19375867231221151 (PMC11080379; doi:10.1177/19375867231221151)
Supplement: Supplemental Material, sj-docx-1-her-10.1177_19375867231221151 - Toward a Future Orientation: A Supportive Mental Health Facility Environment [file sj-docx-1-her-10.1177_19375867231221151.docx]

**Interview guide for initiators, architects, and project managers at Nybygg Psykisk Helse Kristiansand**

What is your role in this project?

What is the main idea behind the new building according to your understanding?

Please tell me about your expectations related to this project and its surroundings.

What is new or different about this building compared to the existing, mental health facility?

In what ways can new possibilities be added to patient care or to the therapeutical work compared to what’s offered currently?

What opportunities does nature offer in connection with hospital stays and treatment?

Describe how you think nature can play a role in the new building?

Is there anything else on your mind about the process around Nybygg Psykisk Helse Kristiansand that we haven't touched on?

Is there anything you would like to ask or add before we close?
